# A remarkable year for NSCLC: Seven new FDA approvals in 2025 across molecular targets

**DOI:** 10.17305/bb.2026.13832

**Published:** 2026-01-05

**Authors:** Krešimir Tomić, Semir Vranić

**Affiliations:** 1 Department of Oncology, University Hospital Center Mostar, Mostar, Bosnia and Herzegovina; 2 College of Medicine, QU Health, Qatar University, Doha, Qatar

**Figure 1.**
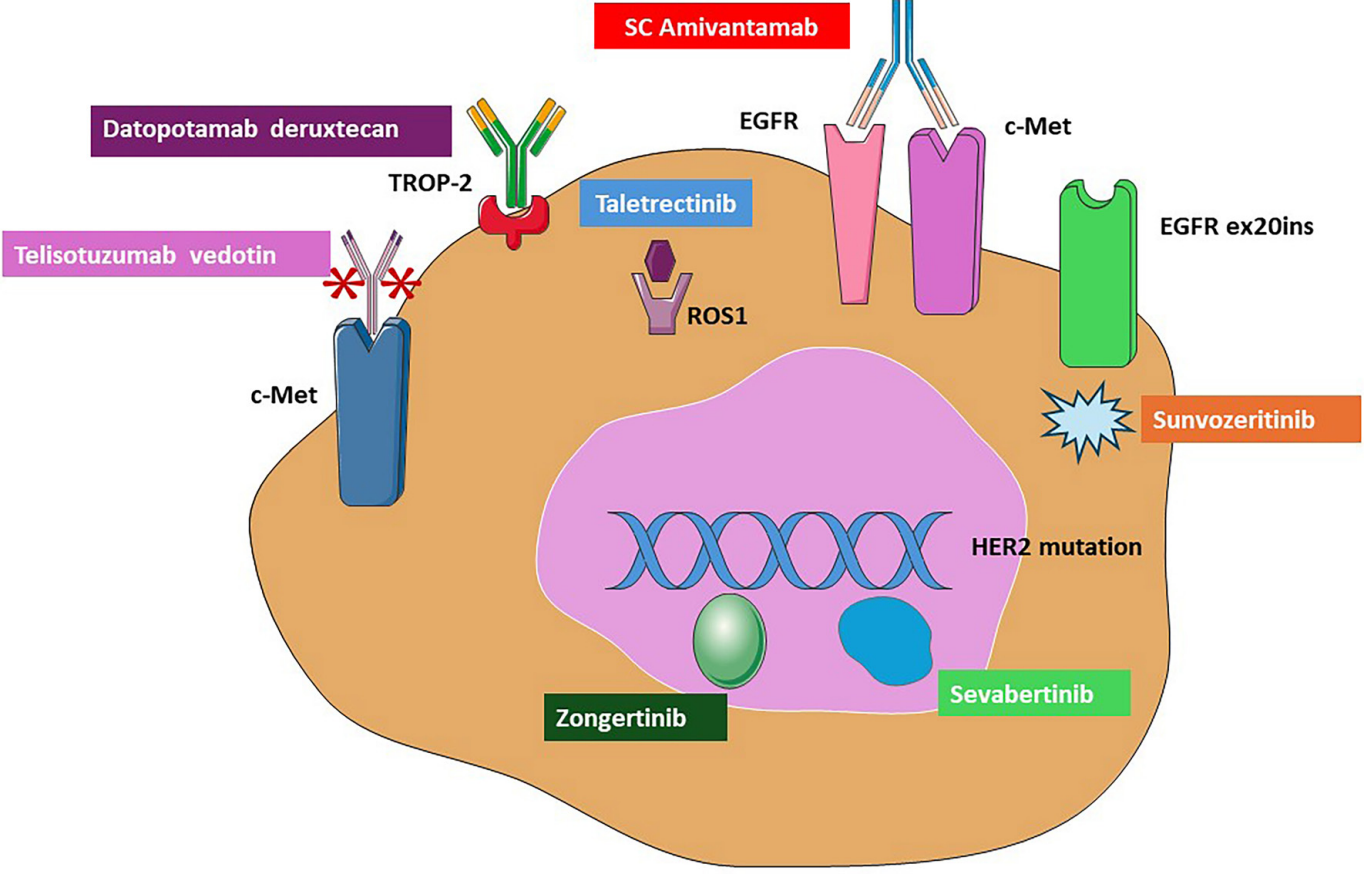
**Molecular targets and corresponding FDA-approved therapies in NSCLC.** The figure illustrates oncogenic drivers in NSCLC and seven recently FDA-approved agents, including antibody–drug conjugates, bispecific antibodies, and tyrosine kinase inhibitors. Together, the figure highlights the molecular heterogeneity of NSCLC and the expanding landscape of biomarker-driven targeted therapies. Abbreviations: FDA: Food and Drug Administration; NSCLC: Non-small cell lung cancer; SC: Subcutaneous; c-MET: Mesenchymal–epithelial transition factor; TROP-2: Trophoblast cell-surface antigen 2; EGFR: Epidermal growth factor receptor; EGFR ex20ins: EGFR exon 20 insertion mutation; HER2: Human epidermal growth factor receptor 2; ROS: ROS proto-oncogene 1. *Image adapted from Servier Medical Art (https://smart.servier.com), licensed under CC BY 4.0 (https://creativecommons.org/licenses/by/4.0/).*

Non-small cell lung cancer (NSCLC) remains the leading cause of cancer-related morbidity and mortality worldwide. However, the past two decades have led to a significant shift in our understanding of the biology of this aggressive disease. The identification of targetable epidermal growth factor receptor (*EGFR)* mutations—an “Achilles heel” in NSCLC—has initiated the era of precision oncology in lung cancer, leading to dramatic improvements in clinical outcomes through the use of tyrosine kinase inhibitors (TKIs). By 2025, the impact of this transformation is underscored by seven new approvals from the U.S. Food and Drug Administration (FDA) for personalized, biomarker-driven targeted therapies in NSCLC (Figure 1). All approvals are based on specific genomic alterations (Figure 1).

The initial approval in May 2025 relates to c-MET, a receptor tyrosine kinase encoded by the MET proto-oncogene (MET) [1]. The approved drug, telisotuzumab vedotin, is a MET-directed antibody-drug conjugate (ADC) indicated for patients with stage IV non-squamous NSCLC exhibiting strong c-MET protein overexpression by immunohistochemistry (IHC 3+ in ≥50% of tumor cells) after prior systemic therapy [2]. This decision further expands the ADC landscape in NSCLC, reinforcing their role as a viable therapeutic class within biomarker-driven treatment strategies. FDA approval was based on findings from the phase II LUMINOSITY study, which demonstrated clinically meaningful objective response rates (ORRs) and durable responses [3]. This approval highlights the clinical value of the ADC approach, enabling precise delivery of cytotoxic agents directly to tumor cells, thereby enhancing efficacy while maintaining manageable toxicity. Importantly, telisotuzumab vedotin redefines the diagnostic algorithm for MET-altered NSCLC: in addition to next-generation sequencing (NGS) for *MET* exon 14 skipping mutations and fluorescence *in situ* hybridization (FISH) for *MET* amplification, IHC assessment of cMET protein overexpression has emerged as a critical biomarker for patient selection.

The expansion of ADCs in NSCLC targeted therapy is further illustrated by the second FDA approval targeting trophoblast cell-surface antigen-2 (TROP-2). TROP-2 expression was initially identified in normal tissues (e.g., trophoblasts and fetal tissues), but its overexpression has since been observed in various cancers [4]. The TROP-2–directed datopotamab deruxtecan (Dato-DXd) received approval for patients with metastatic EGFR-mutated NSCLC who have previously received EGFR-targeted therapy and platinum-based chemotherapy [5]. In the TROPION-Lung 05 (phase II) and TROPION-Lung 01 (phase III) studies, Dato-DXd demonstrated clinically meaningful ORR and median duration of response (mDoR) [6, 7]. However, key questions remain regarding the optimal biomarker (TROP-2 testing by IHC) for Dato-DXd and its ideal position within the treatment sequence for EGFR-mutant NSCLC. This uncertainty is particularly relevant in light of phase III data indicating that first-line combinations of osimertinib plus chemotherapy, or the EGFR-MET bispecific antibody amivantamab combined with the third-generation EGFR inhibitor lazertinib, are superior to osimertinib monotherapy, thereby complicating therapeutic sequencing decisions. As these combination strategies advance earlier in the treatment course, the focus has shifted to optimizing drug delivery without compromising efficacy. In this context, subcutaneous amivantamab, administered alongside oral lazertinib, a third-generation EGFR TKI, has received FDA approval based on the phase III PALOMA study, which demonstrated non-inferior efficacy and a safety profile comparable to intravenous amivantamab combined with lazertinib [8, 9]. The subcutaneous formulation was approved for all indications previously granted for the intravenous formulation. Concurrently, for the rarer but clinically challenging *EGFR* exon 20 insertion mutations, sunvozertinib has emerged as a newly FDA-approved therapeutic option [10]. Based on the phase II WU-KONG1B study, sunvozertinib exhibited clinically meaningful and durable responses following progression on platinum-based chemotherapy, regardless of prior amivantamab exposure [11].

The remaining three FDA approvals in 2025 involve TKIs: one targeting ROS1-rearranged NSCLC and two targeting at human epidermal growth factor receptor 2 (HER2)-altered NSCLC [12–14]. Rearrangements of the c-ros oncogene 1 (ROS1) occur in approximately 1–2% of lung adenocarcinomas and are characterized by significant sensitivity to ROS1 inhibitors [15, 16]. Based on results from the phase II TRUST-I and TRUST-II studies, taletrectinib was approved for both treatment-naïve and previously treated patients [17, 18]. Given its high ORR, central nervous system activity, prolonged progression-free survival (PFS), and efficacy against acquired resistance mutations, taletrectinib, along with repotrectinib, is currently considered a preferred first-line option over entrectinib or crizotinib [19]. The choice between taletrectinib and repotrectinib remains a clinical decision in the absence of head-to-head comparative trials.

HER2-targeted therapy has already transformed the management of HER2-positive breast cancer; however, its application in NSCLC presents complexities due to pronounced biological heterogeneity. In this context, *HER2 (ERBB2)* amplification, mutation, and HER2 protein overexpression represent distinct and variably prevalent alterations, complicating patient selection and therapeutic positioning. Unlike breast and gastric/gastroesophageal junction cancers, anti-HER2 therapy in metastatic NSCLC is currently approved in the second-line setting. Following progression on prior systemic therapy, the FDA has approved three therapeutic options for HER2-mutant NSCLC: the ADC fam-trastuzumab deruxtecan (T-DXd), based on the phase II DESTINY-Lung01 study, and two TKIs with robust efficacy—zongertinib (Beamion LUNG-1) and sevabertinib (SOHO-1) [20, 21]. In contrast to zongertinib and sevabertinib, which require molecular confirmation of HER2 mutations, T-DXd occupies a unique position as the first tumor-agnostic HER2-directed therapy for previously treated patients with metastatic HER2 IHC 3+ solid tumors, including NSCLC, even in the absence of *HER2 (ERBB2)* mutation status [22, 23].

Collectively, these seven FDA approvals in 2025 signify the full maturation of precision oncology in NSCLC, transforming therapeutic decision-making from a one-size-fits-all approach to a strategy defined by tumor biology, molecular profiling (sequencing), and clinical context. The challenge lies in translating these therapeutic advances into accessible and implementable treatments globally.
